# Echocardiographic Parameters to Predict Atrial Fibrillation in Clinical Routine—The EAHsy-AF Risk Score

**DOI:** 10.3389/fcvm.2022.851474

**Published:** 2022-03-08

**Authors:** Jan-Thorben Sieweke, Jan Hagemus, Saskia Biber, Dominik Berliner, Gerrit M. Grosse, Sven Schallhorn, Tobias Jonathan Pfeffer, Anselm A. Derda, Jonas Neuser, Johann Bauersachs, Udo Bavendiek

**Affiliations:** ^1^Department of Cardiology and Angiology, Hannover Medical School, Hannover, Germany; ^2^Department of Neurology, Hannover Medical School, Hannover, Germany

**Keywords:** echocardiography, atrial fibrillation, prediction, atrial remodeling atrial fibrillation, PA-TDI interval, score

## Abstract

**Background:**

Echocardiographic parameters representing impaired left atrial (LA) function and remodeling are of high value to predict atrial fibrillation (AF). This study aimed to develop a prediction model for AF easily to apply in clinical routine containing echocardiographic parameters associated with LA remodeling and—function.

**Methods and Results:**

This monocentric, semi-blinded, controlled analysis included 235 patients to derive a prediction model. This prediction model was tested in a validation cohort encompassing 290 cardiovascular inpatients. The derivation and validation cohort included 54 (23%) and 66 (23%) patients with AF, respectively. Transthoracic echocardiography, comprising parameters indicating left atrial remodeling [septal/lateral total atrial conduction time (s/l PA-TDI)] and left atrial volume indexed to a' (LAVI/a') was performed in each patient. Based on multivariable regressions analysis, four variables were enclosed into the EAHsy (Echocardiography, Age, Hypertension)-AF risk score for AF prediction: Hypertension, Age, LAVI/a‘ and septal PA-TDI. In the validation cohort discrimination was strong (C-statistic 0.987, 95%CI 0.974–0.991) with an adequately performed calibration. The EAHsy-AF risk score was associated with a more precise prediction of AF in comparison to commonly used AF-scores (CHADS_2_-, ATLAS-, ARIC-, CHARGE-AF score).

**Conclusion:**

The EAHsy-AF-Score containing age, hypertension and echocardiographic parameters of atrial dysfunction and remodeling precisely predicts the incidence of AF in a general population of patients with cardiovascular disease. The EAHsy-AF risk score may enable more selective rhythm monitoring in specific patients at high risk for AF.

## Introduction

Early detection of atrial fibrillation (AF) is highly relevant to prevent cardio-embolic stroke and thus preclude death and disability (1). Unfortunately, the diagnosis of AF is frequently delayed because of its clinical non-appearance. Therefore, there is a debate to extend rhythm monitoring in patients after stroke even beyond 72 h as currently recommended by the European Society of Cardiology guidelines (2–4). However, extended ECG monitoring leads to increased healthcare costs, personnel workload, and increased effort for patients (5–8). Therefore, risk evaluation of AF and a step-by-step diagnostic assessment of AF seem to be indispensable.

Due to the importance of AF detection several prediction models have been previously published with different limitations. These risk models are based upon previously collected parameters for AF-prediction or strong associations with AF. AF prediction scores have been developed in patient populations with defined cardiovascular risk (Atherosclerosis Risk in Communities (ARIC) study) and after pulmonary vein isolation (ATLAS) (9, 10). By pooling multiple, in part diverse study populations, the Cohorts for Aging and Research in Genomic Epidemiology (CHARGE)-AF consortium was able to extend the applicability to a broader population compared to the studies mentioned above (11). The CHADS_2_ and CHA_2_DS_2_-VASc scores were designed to assess stroke events in AF. Remarkably, both scores were associated with a known risk of AF and predictive for AF (12).

Onset and maintenance of AF are associated with left atrial (LA) remodeling, dysfunction and fibrosis (13, 14). Total atrial conduction time (PA-TDI interval) and the ratio of LA volume index to tissue Doppler A' (LAVI/ a') both correlate with LA remodeling and are valuable predictors of AF in patients in sinus rhythm (14–16). Recently, we identified echocardiographic parameters, biomarkers and micro RNAs indicating patients at high risk for AF, which potentially enables risk-stratified decision making (15, 17).

In the present analysis, we aimed to improve AF prediction models by including echocardiographic parameters associated with LA remodeling and dysfunction easily obtained in clinical routine. These findings may enable a more specific selection for extended rhythm monitoring in patients at high risk for AF.

## Methods

### Study Design and Participants

The study was conducted at a single center and had a semi-blinded and controlled design. In particular, blinded investigators unaware of the AF status performed echocardiographic analysis. Furthermore, investigators unaware of echocardiographic analysis performed evaluation of the ECG. It was approved by the local ethics committee of Hannover Medical School (application number: 3316-2016 and 8276_BO_K_2019) and complied with the Declaration of Helsinki.

All participants underwent a 12-lead electrocardiogram (ECG), comprehensive transthoracic echocardiography and at least had one long-term ECG in the former history. Exclusion criteria were defined as follows: age <18 years, severe mitral valve stenosis or regurgitation, history of aortic or mitral valve replacement, ablation of supraventricular tachycardia, history of cardiac surgery, class I antiarrhythmic therapy and patients, unable to provide informed consent.

Between 1st August 2016 and 31st August 2017, consecutive patients presenting in sinus rhythm were included in the derivation cohort: (1) Patients admitted to our local Stroke Unit, the cardiology ward and a group of volunteers without documented AF (∑n = 175), who were formerly included in a separate clinical study (15) and (2) patients with cardiovascular diseases regularly treated in the Department of Cardiology and Angiology of Hannover Medical School after implementing septal PA-TDI and LAVI/a' in clinical routine (∑n = 60). The validation cohort consists of cardiovascular inpatients in sinus rhythm screened from 10/2017 to 04/2019 (∑n = 290). In the validation cohort patients had a 24 h-Holter-ECG monitoring. Furthermore, during the in-hospital stay all patients had a telemetric ECG-monitor surveillance for at least 48 h.

All data were obtained from patients in clinical routine, except for a part of patients in the derivation cohort (*n* = 175).

### Echocardiography

Data of transthoracic echocardiography were collected according to the American Society of Echocardiography guidelines and were obtained in clinical routine in the validation cohort and a portion of the derivation cohort (18). As chosen parameters for LA remodeling cannot be obtained in the presence of AF, only patients in sinus rhythm were considered in this study.

Echocardiographic parameters of LA-function and—remodeling were determined as recently described in the left lateral decubitus during brief breath hold (15, 17). In brief, septal PA-TDI was defined as the interval between the onset of P-wave in lead II of the ECG on echocardiographic images and the peak A'-wave of the septal mitral valve (MV) annulus in tissue Doppler imaging. LA volumes were assessed at the ventricular end-systole according to the recommendations of the American Society of Echocardiography and the European Association of Cardiovascular Imaging (19) by biplane area length method in apical 4- and 2-chamber views. Subsequently, after LA volume was indexed to body surface area, LAVI/a' was determined with the average of septal and lateral a'.

### ECG Examination

On the basis of ECG findings AF was diagnosed. All participants in the derivation and in the validation-cohort had a 12-channel surface ECG. ECG examination of the derivation cohort was previously described (15). In brief, patients with acute stroke were monitored with a two-channel Holter-ECG-monitoring (GE Healthcare SEERTM 1000, Great Britain) scheduled for 72 h. In the validation cohort patients with a history of AF and/ or documented AF in a 24 h-LT-ECG were included in the cohort of patients with AF and vice versa. In the validation cohort 24 h-LT-ECG was performed in clinical routine in the out-patient-clinic or at the general practitioner.

All ECG recordings were assessed offline using analysis software (CardioDay; getemed Medizin- und Informationstechnik) by professionals, who were blinded to echocardiographic and clinical data, taking into account the diagnostic criteria of current guidelines on AF (20).

### Statistical Analysis

Statistical analysis and graphical presentation were performed using SPSS Statistics 26 (IBM SPSS Statistics 26) and GraphPad Prism 7.04 (Graph Pad Software, San Diego, CA, USA). Results are presented as numbers (n) and percentages (%) for categorical variables and for continuous variables either as mean ± standard deviation (SD) for quantification of normal distribution or median and interquartile ranges (IQR) for non-normally distributed variables. Normality and variance homogeneity were checked by Shapiro-Wilk and D‘Agostino Pearson test. Comparisons between groups were performed using Student's *t*-test for Gaussian distributed data and the Mann-Whitney test in non-normally distributed data. Categorical variables were evaluated by χ^2^ test. A two-sided *P*-value of < 0.05 was considered statistically significant.

Logistic regression analysis was performed to identify parameters associated with AF. Univariable regression analysis was performed including all variables potentially associated with AF (*p* < 0.05). Subsequently, after testing for multicolinearities, predictors of AF were determined using a stepwise backwards multivariable regression analysis with variables, which significantly linked to AF in univariable analysis (*p* < 0.1). Results from the regression analyses are presented as hazard ratios (HRs) with 95% confidence intervals (CIs), respectively.

The risk prediction model for AF was constructed in a derivation cohort and validated in an internal cohort. The AF prediction model was constructed based on parameters which were independently associated with AF (septal PA-TDI, LAVI/a'), previously reported to be independently associated [hypertension (9, 11, 21, 22)] and/or tightly missed significance (age (9, 21, 22). Only patients with complete datasets for candidate variables were considered for further testing.

To increase its clinical applicability and exclude limitations due to restricted software, we used echocardiographic parameters easily to be assessed in clinical routine. Furthermore, we used categorical parameters (age >75 years, previous history of hypertension) and dichotomized metric parameters (septal PA-TDI >121 ms and LAVI/a'>3.3) to simplify applicability of these parameters in the score. To create a differentiation model between patients with and without AF separating values were chosen based on cut-off values with 95% confidence interval and subsequent determination of the odds ratio (e.g., septal PA-TDI: <121 ms OR: 1.72 95%CI 0.41–7.12; >121 ms OR: 14.5 95%CI 5.63–37.24).

In the derivation dataset, regression coefficients (β) of independent predictors (septal PA-TDI and LAVI/a') were used to estimate and weight the predictive impact of the variables, assigning one score point per β of 0.1 (0.05–0.149) (23).

The discriminative ability of the risk prediction model was assessed by the area under the receiver operating characteristic (ROC) curve, and the final score determined in the validation cohort was compared with previously published scoring systems: ATLAS (10), ARIC (9), CHARGE (11), CHADS_2_ (24). Additionally, Youden's index was determined to ascertain cut-off values of the AF scores. Subsequently, under assumption of a dichotomous distribution using cut-off values, the final score and the previously published prediction models were compared by McNemar test.

The distribution of the population and predicted and observed AF diagnosis were calculated.

## Results

### Characteristics of the Derivation Cohort

Between 08/2016 and 08/2017, 235 patients with a median age of 65 [IQR 52–75] years (62% males) and a median left ventricular ejection fraction of 60% [IQR 56–64] were included in the derivation cohort. As a part of the derivation cohort, 175 patients were described in a recently published pilot-study, which identified echocardiographic parameters as predictors for AF in patients with embolic stroke of undetermined source and controls (15). Stroke-Classification was performed before Holter ECG-Monitoring. A total of 115 patients with acute cerebral infarction were included in the study. Of these, 69 patients were categorized into the group with embolic stroke of undetermined source (ESUS), 5 patients with pre-existing chronic paroxysmal AF, 16 patients with macrovascular stroke and 25 patients with microvascular stroke. The median National Institutes of Health Stroke Scale (NIHSS) score was 2 (IQR: 1–4). Patients in the stroke cohort had cardiovascular risk factors (hypertension 72%, diabetes 22%, current smoking 27%). In addition, the derivation cohort included patients with chronic paroxysmal AF without acute stroke (*n* = 36), participants without documented AF, cardiovascular disease, or acute stroke (*n* = 36), patients with symptomatic coronary artery disease (*n* = 48, of whom *n* = 15 had peripheral arterial disease). Baseline characteristics and echocardiographic parameters of patients with and without AF in the derivation cohort are listed in Table 1 and Supplementary Table 1. Paroxysmal AF was diagnosed in 54 (23%) patients with a median duration of AF-episodes of 78 s [47–196 s]. Patients with AF were significantly older and hypertension was more frequent. In the derivation cohort, no patients were found to have hyperthyroidism and channelopathies as a pre-existing condition. Significant differences were observed between patients with and without AF for echocardiographic parameters indicating left atrial function and remodeling: LAVI/a', lateral atrial conduction time (lateral PA-TDI), and septal atrial conduction time (septal PA-TDI).

**Table 1:** Baseline characteristics and transthoracic echocardiography at inclusion of the derivation and validation cohort.

**Parameter**	**Derivation Cohort**				**Validation Cohort**			
	**All**	**w/o AF**	**AF**	**P w/o AF vs. AF**	**All**	**w/o AF**	**AF**	**P w/o AF vs. AF**
	***n =* 235**	***n =* 181**	***n =* 54**		***n =* 290**	***n =* 224**	***n =* 66**	
Age [years]	65 [52–75]	62 [49–74]	72 [65–78]	***<0.001***	59.5 [45–71]	54 [39.3–67]	70 [65–77]	***<0.001***
Sex:Female	88 (37.4%)	69 (38.1%)	19 (35.2%)	0.696	121 (41.7%)	89 (39.7%)	32 (48.5%)	0.205
**Pre-existing conditions**								
Hypertension	144 (61.3%)	100 (55.2%)	44 (81.5%)	***0.001***	168 (57.9%)	116 (51.8%)	52 (78.8%)	***<0.001***
Diabetes	35 (14.9%)	26 (14.4%)	9 (16.7%)	0.677	33 (11.4%)	23 (10.3%)	10 (15.2%)	0.272
Stroke	41 (17.5%)	32 (17.7%)	9 (16.8%)	0.067	17 (5.9%)	10 (4.5%)	7 (10.6)	0.062
Current smoking	65 (27.7%)	54 (29.8%)	11 (20.4%)	0.172	94 (32.4%)	77 (34.4%)	17 (25.8%)	0.352
Alcohol abuse	0	0	0		0	0	0	
CHADS_2_	1 [0–2]	1 [0–2]	2 [1–2]	***0.007***	1 [0–2]	1 [0–1]	1 [1–2]	***<0.001***
0	66 (28.1%)	60 (33.1%)	6 (11.1%)		94 (32.4%)	86 (38.4%)	8 (12.1%)	
1	72 (30.6%)	53 (29.3%)	19 (35.2%)		120 (41.4%)	87 (38.8%)	33 (50%)	
2	50 (21.3%)	33 (18.2%)	17 (31.5%)		55 (19%)	42 (18.8%)	13 (19.7%)	
3	30 (12.8%)	24 (13.3%)	6 (11.1%)		16 (5.5%)	8 (3.6%)	8 (12.1%)	
4	12 (5.1%)	9 (5%)	3 (5.6%)		4 (1.4%)	1 (0.4%)	3 (4.5%)	
5	5 (2.1%)	2 (1.2%)	3 (5.6%)		1 (0.3%)	0	1 (1.5%)	
CHA_2_DS_2_-VASc	2 [1–4]	2 [1–4]	3 [2–4]	0.074	2 [1–3]	2 [1–3]	3 [2–4]	***<0.001***
0	39 (16.6%)	35 (19.3%)	4 (7.4%)		37 (12.8%)	32 (14.3%)	5 (7.6%)	
1	44 (18.7%)	36 (19.9%)	8 (14.8%)		64 (22.1%)	60 (26.8%)	4 (6.1%)	
2	42 (17.9%)	35 (19.3%)	7 (13%)		69 (23.8%)	54 (24.1%)	15 (22.7%)	
3	37 (15.7%)	27 (14.9%)	10 (18.5%)		49 (16.9%)	36 (16.1%)	13 (19.7%)	
4	39 (16.6%)	26 (14.4%)	13 (24.1%)		42 (14.5%)	28 (12.5%)	14 (21.2%)	
5	24 (10.2%)	15 (8.3%)	9 (16.7%)		20 (6.9%)	12 (5.4%)	8 (12.1%)	
6	6 (2.6%)	5 (2.8%)	1 (1.9%)		8 (2.8%)	2 (0.8%)	6 (9.1%)	
7	3 (1.3%)	2 (1.2%)	2 (3.7%)		0	0	0	
8	1 (0.4%)				1 (0.3%)	0	1 (1.5%)	
LVEF [%]	60 [56–64]	60 [56–64]	59 [54–63]	0.169	55 [49.2–56.9]	55 [48–57]	55 [50.5–56.4]	0.746
PA-TDI septal [ms]	98.3 [85.0–118.0]	90.7 [81.5–101.0]	135.6 [131.0–142.0]	***<0.001***	103.5 [85–119]	97.3 [82.6–110.4]	130 [126–142.1]	***<0.001***
PA-TDI lateral [ms]	109.0 [97.5–126.0]	104.0 [94.0–113.8]	146.9 [137.8–158.1]	***<0.001***	114 [100.5–131.5]	111 [98–124]	145 [127–158]	***<0.001***
LAVI/a'	3.3 [2.8–4.2]	3.2 [2.6–3.8]	4.2 [3.4–7.1]	***<0.001***	3.4 [2.6–4.5]	3.1 [2.4–4.1]	4.5 [3.7–6.9]	***<0.001***

*LAVI, left atrial volume indexed to body surface area; LVEF, left ventricular ejection fraction; PA-TDI, total atrial conduction time interval. P-values < 0.05 are displayed by bold characters*.

### Derivation of the EAHsy-AF Risk Score

In the derivation cohort consisting of 235 patients, multivariable regression analysis identified septal PA-TDI [ms] [HR 1.41 (95%CI 1.21–1.64), *p* < 0.001)], and LAVI/a' [HR 1.81 (95%CI 1.43–2.31), *p* = 0.007)] as independent predictors of AF presence (Supplementary Table 2). To account for individual predictive weight in the AF-Score, one score point was assigned per regression coefficient (β) in the multivariable model to identify the maximal point range. Therefore, the maximal point for septal PA-TDI (β = 0.375) was 4 and for LAVI/a' (β = 0.159) was 2 points. In addition, the parameters hypertension and age were considered because of their independent association with AF demonstrated in previous studies, although they missed significance in the present analysis. These parameters were weighted with one point according to the ratio of the specific odds ratio. Finally, four parameters were included in EAHsy (Echocardiography, Age, Hypertension)-AF risk score. All points of the four variables were used for total score calculation, giving a maximum of eight points.

**Figure 1:**
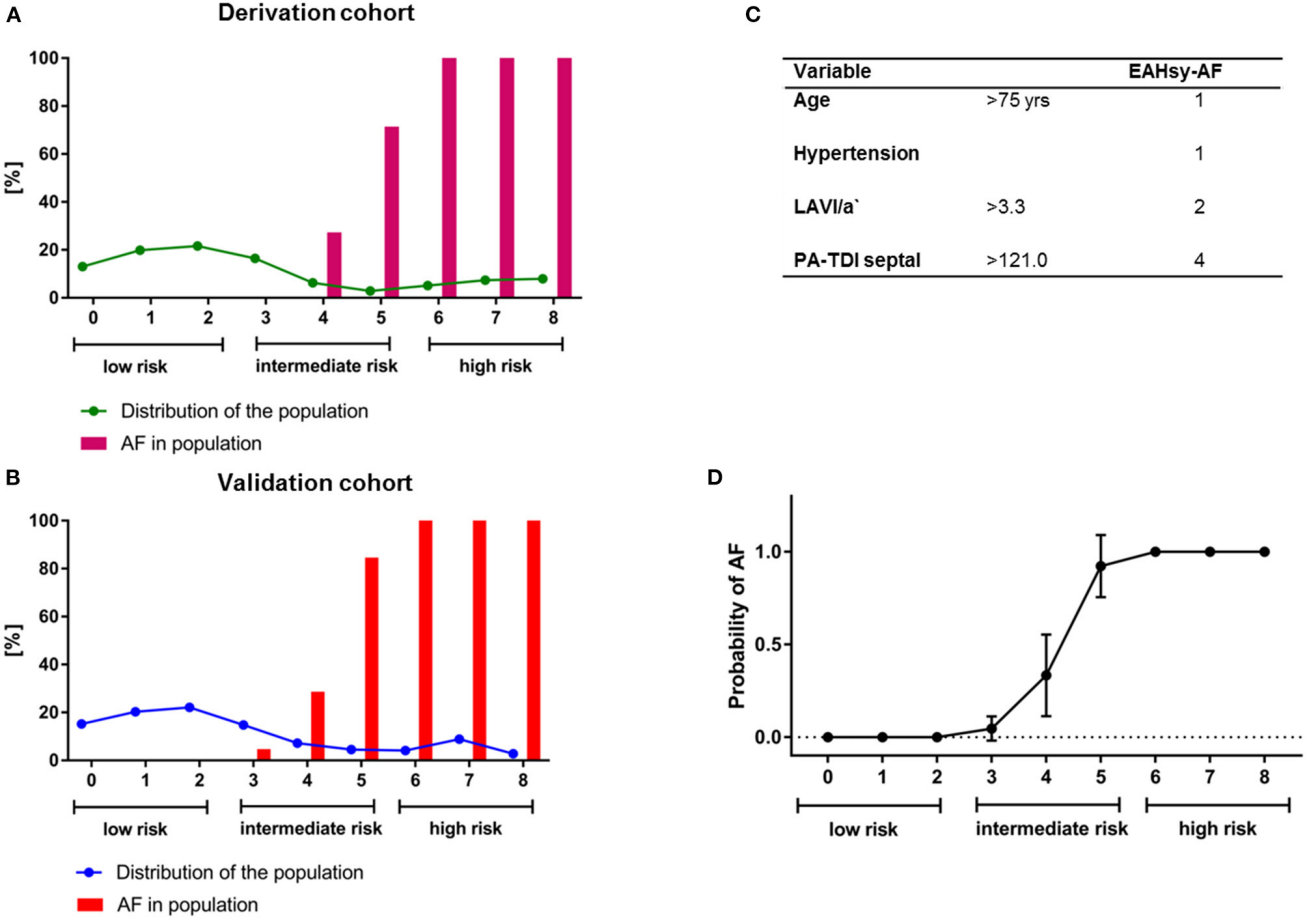
Distribution of the population (line) and diagnosis of AF (%; bars) according to the cumulative points from the EAHsy AF-Score in the derivation **(A)** and validation cohort **(B)**. The EAHsy-AF score consists of 4 parameters with a maximum of cumulative points of 8 **(C)**. The population can be classified according to the EAHsy- AF-Score into low (scores 0–2 pts), intermediate (scores 3–5 pts), and high risk (scores 6–8 pts) with an observed diagnosis of AF. Symbols show the mean with 95%CI. The x-axis depicts the cumulative points from the EAHsy-AF-Score, and the y-axis the probability of AF **(D)**. AF, atrial fibrillation.

The prediction of AF was positively correlated with the sum of the total individual score. Risk categories were defined: low risk (0–2 points), intermediate risk (3–5 points), high risk (6–8 points). Regression analysis and variables of the AF-Score with their corresponding score points are provided in Supplementary Table 2. Distribution of the population and diagnosis of AF according to the cumulative points from the EAHsy -AF risk score are provided in Figure 1.

### Validation of the EAHsy-AF Risk Score

In the validation cohort, 318 patients were screened for eligibility. The prediction model was validated in a cohort of 290 patients (59% males) with a median age of 60 years [IQR 45–71] and a median left ventricular ejection fraction of 55% [IQR 49–57]. At initial medical consultation in the validation cohort, the following reasons for the patient presentation were documented: symptomatic coronary artery disease (35%), cardiac arrhythmias (22%), cardiomyopathies (22%), arterial hypertension (11%), syncope (4%), pulmonary artery embolism (2%), persistent foramen ovale (2%), and deep vein thrombosis (2%). In the validation cohort, 66 patients (23%) had confirmed AF. Between patients with and without AF, age and hypertension were significantly altered. Furthermore, echocardiographic parameters of diastolic function (MV E/e' septal/lateral), atrial dimension (LAVI, RAVI) and LA remodeling and function (LAVI/a', lateral/septal PA-TDI) were statistically significantly different between patients with and without AF (Table 1 and Supplementary Table 1). Like the derivation cohort, the validation cohort did not include patients with channelopathies or hyperthyroidism.

Multivariable regressions analysis in the validation cohort confirmed septal PA-TDI and LAVI/a' as independent predictors for AF (Supplementary Table 3). After replacing these two parameters with the prediction model, the AF score was an independent predictor in the multivariable regressions analysis as shown in Table 2. Distribution of the population and diagnosis of AF according to the cumulative points from the EAHsy-AF risk score and the increase in predicted and observed AF frequency with higher cumulative score points are provided in Figure 1.

**Table 2:** Predictor of AF in multivariate regressions analysis in the validation cohort.

**Parameter**	**Complete Cohort (*n =*** **290)**		**Complete Cohort (*n =*** **290)**	
	**Univariate regression analysis**		**Multivariate regression analysis**	
	**HR (95%CI)**	***p*-value**	**HR (95%CI)**	***p*-value**
Age	1.078 (1.052–1.105)	***<0.001***		
Height	0.965 (0.94–0.991)	***0.009***	0.929 (0.813–1.061)	0.276
Hypertension	3.458 (1.813–6.596)	***<0.001***		
CHADS_2_	1.928 (1.44–2.582)	***<0.001***		
CHA_2_DS_2_-VASc	1.593 (1.324–1.916)	***<0.001***	0.079 (0.001–7.326)	0.272
EAHsy-AF risk score	25 (6.518–95.943)	***<0.001***	208.972 (6.718–6499.994)	***0.002***
LAVI	1.069 (1.043–1.097)	***<0.001***	0.965 (0.818–1.139)	0.677
RAVI	1.25 (1.121–1.394)	***<0.001***	1.510 (0.59–3.866)	0.391
PA-TDI septal	1.217 (1.15–1.288)	***<0.001***		
PA-TDI lateral	1.045 (1.031–1.059)	***<0.001***	0.904 (0.913–1.005)	0.061
LAVI/a'	1.357 (1.195–1.54)	***<0.001***		
MV E/e'septal	1.141 (1.078–1.208)	***<0.001***	0.935 (0.483–1.81)	0.842
MV E/e'lateral	1.122 (1.056–1.192)	***<0.001***	0.832 (0.663–1.044)	0.113

*AF, atrial fibrillation; CI, confidence interval; HR, hazard ratio; LAVI, left atrial volume indexed to body surface area; MV, mitral valve; PA-TDI, total atrial conduction time interval; RAVI, right atrial volume indexed to body surface area. P-values < 0.05 are displayed by bold characters*.

**Figure 2:**
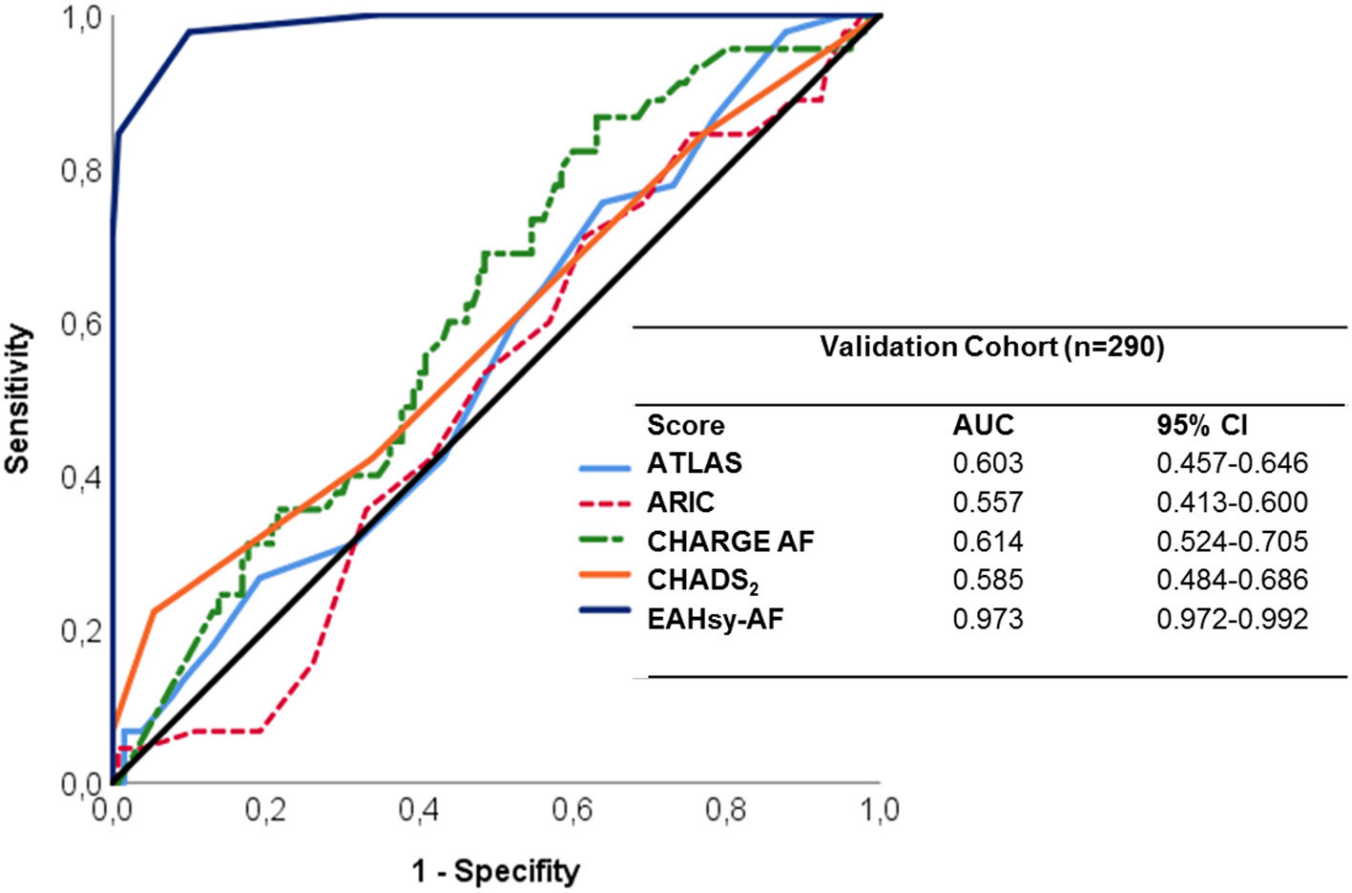
ROC-Curves analysis of scores predicting AF in the validation cohort.

The EAHsy-AF risk score exhibited strong discriminability with an AUC of 0.97 (95%CI 0.97–0.99) as provided in Figure 2.

### Comparison of the EAHsy-AF Risk Score to Other Scores Predicting AF

The AUC and separation values were individually determined for the EAHsy-AF risk score and comparators in the derivation and validation cohort to identify the influence of statistical outliers. All used scores had consistent separation and AUC values. In both cohorts, the EAHsy-AF risk score had the best accuracy in discriminating patients with and without AF in the derivation, validation and complete cohort (Supplementary Table 4 and Figure 2). For comparison, in the McNemar test using the individually determined cut-off values, the superior diagnostic value of the EAHsy-AF risk score was confirmed compared to CHADS_2_-, CHARGE AF-, ARIC-, and ATLAS-Score as presented in Supplementary Table 5.

## Discussion

Clinically silent AF is often underdiagnosed. Therefore, identifying patients at high risk for AF is of tremendous importance (25). Left atrial fibrosis and—remodeling are known causes of AF and correlate with echocardiographic parameters. The ratio of indexed LA volume and mitral annulus velocity during atrial contraction (LAVI/a') was recently identified as predictors of AF (14–16). Additionally, we identified the echocardiographic parameter septal PA-TDI, a predictor of subclinical AF in patients with embolic stroke of unknown origin (15).

**Figure 3:**
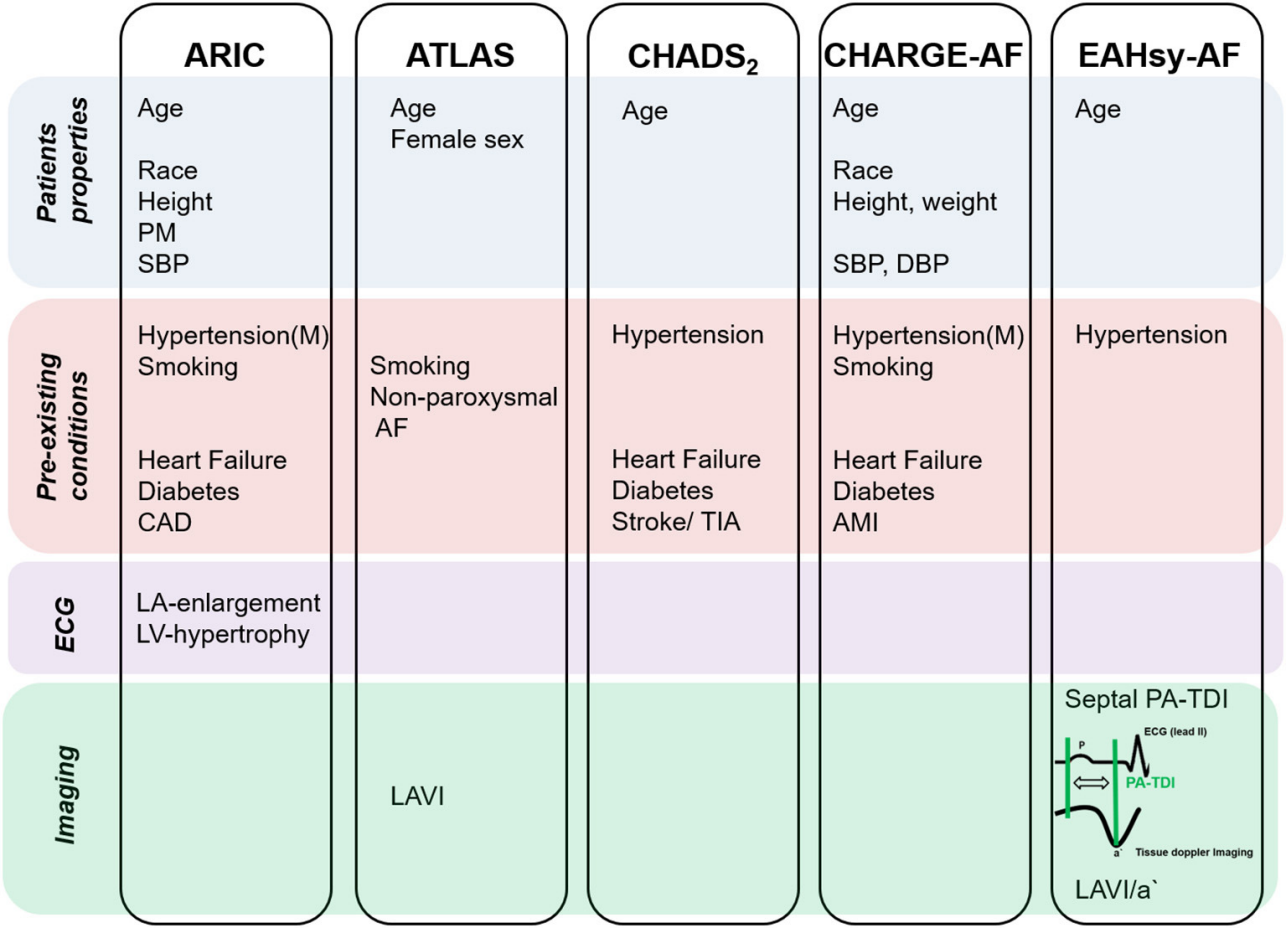
Parameters chosen in scores to predict AF. AF, atrial fibrillation; AMI, acute myocardial infarction; CAD, coronary artery disease; DBP, diastolic blood pressure; Hypertension (M), hypertension medication; LA, left atrial; LAVI, left atrial volume indexed; LV, left ventricular; PM, precordial murmur; SBP, systolic blood pressure; TIA, transient ischemic attack.

In the present study we developed and validated a score depending on patients' characteristics and echocardiographic parameters to identify patients at high and low risk of AF. Various former studies aimed to identify patients with AF. However, some scores were developed based on patients included in clinical trials, such as ATLAS, ARIC and CHARGE-AF (9–11). Other, like the CHADS_2_- and CHA_2_DS_2_-VASc-Score, were developed to predict thromboembolic events, i.e., stroke, in patients with AF and are recommended by current guidelines to clarify an indication for anticoagulation (21, 24, 26) (Figure 3). Although the latter risk models were created with a different hypothesis, both scores have been used for the prediction of AF (12, 27). However, the CHA_2_DS_2_-VASc-Score seems to be inferior to the CHARGE-AF Score in the precision of the prediction of AF (28). In our analysis, the CHARGE-AF Score showed a higher accuracy in the prediction of AF compared to the CHADS_2_- and CHA_2_DS_2_-VASc-Score, too. The CHARGE-AF Score (11) was derived from three studies (Framingham Heart Study, Cardiovascular Health Study, and Atherosclerosis Risk in Communities Study), was validated in two more studies (Rotterdam- and AGES Study) and contains among others the parameters age and arterial hypertension. Both parameters are known risk factors for AF and are included in the ARIC score (9). Therefore, we considered age and arterial hypertension as parameters for the risk prediction model, although both parameters are univariable predictors but not independent predictors in our analysis. In addition, these parameters can efficiently and reliably be determined in clinical routine. A score to predict AF recurrence after radiofrequency pulmonary vein isolation is the ATLAS score (10). The ATLAS- and ARIC-AF Score considered parameters of left atrial dimension (LAVI, LA-enlargement). Beyond the left atrial dimension, LA-function and—remodeling parameters were recently identified as predictors of AF (14–16). In our multivariable analysis of the validation cohort septal PA-TDI and LAVI/a' were confirmed as independent predictors of AF (Supplementary Table 3). Subsequently, we developed a score considering: age, hypertension, LAVI/a' and septal PA-TDI. Frequency of AF-diagnosis correlated with higher cumulative score points from the EAHsy (Echocardiography, Age, Hypertension)-AF risk score.

We calculated previously published scores (ATLAS, ARIC, and CHARGE-AF) and the CHADS_2_-Score in our derivation and validation cohort to determine whether the inclusion of specific echocardiographic parameters of LA-dysfunction and—remodeling within the AF Score is superior to these scores in the ability to discriminate patients with and without AF. All applied scores had a good precision for the discrimination of AF in the validation cohort (Figure 2). Importantly, the CHARGE-AF Score had the highest discrimination ability of the previously published scores (AUC: 0.614) in our validation cohort, which is in line with results of a meta-analysis demonstrating CHARGE-AF to be the most robust of 21 AF prediction models in performance and applicability (29). However, the EAHsy-AF risk score, including specific echocardiographic parameters of LA-dysfunction and –remodeling, was much superior in its discriminative power compared to previously published scores (AUC: 0.973) and in its predictive accuracy confirmed by McNemar testing (Supplementary Table 5). A score that can be collected in clinical routine is the C_2_HEST score (22). This score also showed good discrimination between patients with and without AF in our cohort. However, this score also showed a lower accuracy compared to the EAHsy AF score (AUC in validation cohort: 0.657 CI 0.585–0.729).

The strength of the presented EAHsy-AF risk score is that it can be calculated by few parameters, which can be easily determined in routine cardiologic work-up. The results of our analyses depicted a stepwise increase in AF detection according to the different score categories.

In the prediction of inapparent AF, 12-lead ECG has an important value. PR-interval is also associated with myocardial structural remodeling and AF (30). However, in our previous analysis, this parameter was not independently associated with the presence of AF and was therefore not considered for building this score. Of note, new parameters have recently gained considerable attention. In patients with cryptogenic stroke P-wave dispersion (PWD) and P-terminal force in the precordial lead V1 (PTFV1) are associated with AF (31, 32). However, to increase the clinical applicability and exclude limitations due to restricted software, we would not have included these ECG parameters. Therefore, this score did not consider the parameters of longitudinal strain- and strain rate analyses of the left atrium predicting AF.

Implementing echocardiographic parameters reflecting LA-remodeling and—function, i.e., septal PA-TDI and LAVI/a', in a score for the prediction of AF seems to be of high value. Of importance, these echocardiographic parameters appear to reflect underlying pathophysiological changes in the LA, thereby improving the EAHsy-AF score in its superior ability to predict AF, when compared with other prediction models. Left atrial function and structural remodeling—in particular fibrosis- reflected by gadolinium enhancement in MRI correlate with the echocardiographic parameters lateral total atrial conduction time (Lateral PA-TDI interval) and LAVI/a' (13, 33, 34). Of note, the echocardiographic parameter septal PA-TDI seems to be of particular importance. One possible explanation is that the disturbances of electromechanical coupling, influenced by LA fibrosis as arrhythmic substrate and mapped by PA-TDI, is an essential pathophysiological correlate for the occurrence of AF.

Beyond underlying pathophysiological structural changes of the LA and different prediction models, variations in conduction such as channelopathies, shifts in electrolytes, modulation of the vagal tone or pulmonary vein ectopy due to lifestyle factors lead to the occurrence of AF (35–39). In the analysis of the EAHsy-AF cohort, no patient showed hyperthyroidism, alcohol abuse, channelopathies or performed regular extreme endurance sports. These factors should be considered in addition to the echocardiographic parameters described to prevent AF recurrence.

In this study, limitations should be considered: (1) This study included a relatively small sample size. However, the rigorous diagnostic process with a comprehensive echocardiographic protocol, ECG-monitoring, and the inclusion of patients with stroke strengthen this study. (2) In the current study, the patient cohort includes a higher proportion of patients without atrial fibrillation than patients with atrial fibrillation. Therefore, this score may be more suitable for the exclusion of atrial fibrillation than for its detection. (3) A selection bias owing to solely restriction on parameters of baseline characteristics and echocardiography should be considered. However, our analysis clearly shows that echocardiographic parameters of LA function and remodeling should be taken into account when generating an AF score. (4) External validation of the score would have strengthened its statement and applicability. (5) Clinically silent episodes of AF may be underdiagnosed because of study design.

## Summary/Conclusions

Atrial fibrillation is a main cause of cardio-embolic events and in particular strokes, subsequently provoking functional limitations and death. Besides age and hypertension, the EAHsy-AF risk score contains septal PA-TDI and LAVI/a' as echocardiographic parameters reflecting LA-function and—remodeling. Validated in clinical routine, the easily determined EAHsy-AF risk score seems to be of high value for the discrimination of patients with and without AF in a general population of patients with cardiovascular disease and may enable more selective rhythm monitoring in specific patients at high risk for AF.

## Data Availability Statement

The original contributions presented in the study are included in the article/Supplementary Material, further inquiries can be directed to the corresponding author/s.

## Ethics Statement

The studies involving human participants were reviewed and approved by Ethics Committee of Hannover Medical School. The patients/participants provided their written informed consent to participate in this study.

## Author Contributions

Conceptualization of the analysis were done by J-TS and UB. J-TS, JH, DB, GG, JB, and UB wrote the manuscript. J-TS, JH, SB, DB, GG, SS, TP, AD, JN, JB, and UB revised the manuscript critically. J-TS, JH, and SS edited the final draft. J-TS, JB, and UB accurately approved the manuscript. All authors acquired an analyzed data and agreed to be accountable for all aspects of the work ensuring that questions related to the accuracy or integrity of any part of the work are appropriately investigated and resolved.

## Funding

SB was supported by the Else-Kröner-Fresenius-Stiftung as part of the KlinStrucMed-Program at Hannover Medical School. This work was supported by PRACTIS – Clinician Scientist Program of Hannover Medical School, funded by the German Research Foundation (DFG, ME 3696/3-1) to GG and AD.

## Conflict of Interest

The authors declare that the research was conducted in the absence of any commercial or financial relationships that could be construed as a potential conflict of interest.

## Publisher's Note

All claims expressed in this article are solely those of the authors and do not necessarily represent those of their affiliated organizations, or those of the publisher, the editors and the reviewers. Any product that may be evaluated in this article, or claim that may be made by its manufacturer, is not guaranteed or endorsed by the publisher.

## Glossary

AF: Atrial fibrillation
ATLAS: A-age, T-type of AF, LA-LA-volume, S-Sex and Smoking
CHARGE: Cohorts for Heart and Aging Research in Genomic Epidemiology
ARIC: Atherosclerosis Risk in Communities
Cis: Confidence intervals
EAHsy: Echocardiography, Age, Hypertension
HR: Hazard ratio
LA: Left atrial
LAVI/a': Ratio of LA volume index to tissue Doppler a'
PA-TDI: Total atrial conduction time
ROC: Receiver operating characteristic
