# Supplementary material for: Echocardiographic Parameters to Predict Atrial Fibrillation in Clinical Routine—The EAHsy-AF Risk Score
Source: Front Cardiovasc Med. 2022 Mar 8;9:851474. doi: 10.3389/fcvm.2022.851474 (PMC8957789; doi:10.3389/fcvm.2022.851474)
Supplement: Supplementary file 1 [file Data_Sheet_1.docx]

**Supplemental Table 1: Baseline characteristics and transthoracic echocardiography at inclusion DERIVATION & VALIDATION COHORT**

| **Parameter** | | **Derivation Cohort** | | | | **Validation Cohort** | | | |
| --- | --- | --- | --- | --- | --- | --- | --- | --- | --- |
|  |  | **All** | **w/o AF** | **AF** | **P**  **w/o AF vs AF** | **All** | **w/o AF** | **AF** | **P**  **w/o AF vs AF** |
|  |  | n=235 | n=181 | n=54 |  | n=290 | n=224 | n=66 |  |
| Height [cm] | | 174 [167-179] | 175 [168-180] | 172 [165-177] | 0.071 | 173.5±10.7 | 174.4±9.8 | 170.5±12.7 | ***0.007*** |
| Weight [cm] | | 79 [70-90] | 78 [70-90] | 80 [69-92] | 0.736 | 81 [67-94] | 81 [69-94] | 80 [66-95] | 0.81 |
| Pre-existing conditions | |  |  |  |  |  |  |  |  |
|  | Smoking | 65 (27.7%) | 54 (29.8%) | 11 (20.4%) | 0.172 | 94 (32.4%) | 77 (34.4%) | 17 (25.8%) | 0.352 |
|  | MI | 17 (7.2%) | 10 (5.5%) | 7 (13.0%) | 0.064 | 41 (14.1%) | 28 (12.5%) | 13 (19.7%) | 0.140 |
|  | CAD | 48 (20.4%) | 31 (17.1%) | 17 (31.5%) | ***0.022*** | 125 (43.1%) | 95 (42.4%) | 30 (45.5%) | 0.592 |
|  | PAD | 15 (6.4%) | 10 (5.5%) | 5 (9.3%) | 0.325 | 21 (7.2%) | 17 (7.6%) | 4 (6.1%) | 0.674 |

| LVEDD in 4 CV [cm] | 4.9 [4.6-5.3] | 4.9 [4.6-5.2] | 5.1 [4.7-5.4] | 0.541 | 4.9 [4.6-5.3] | 4.9 [4.6-5.2] | 5 [4.6-5.4] | 0.673 |
| --- | --- | --- | --- | --- | --- | --- | --- | --- |
| RVEDD in 4CV [cm] | 3.6±0.6 | 3.6±0.6 | 3.6±0.6 | 0.548 | 3.6 [3.1-4] | 3.6 [3.1-4] | 3.6 [3.1-3.9] | 0.600 |
| LAVI | 37.5 [30.0-52.9] | 34.4 [28.6-45.6] | 47.1[38.5-70.1] | ***<0.001*** | 31.9 [26.3-41.5] | 29.5 [25.3-38.1] | 40.4 [34.5-46.9] | ***<0.001*** |
| RAVI | 9.8 [7.4-10.7] | 9.5 [7.4-9.8] | 11.3 [7.8-12.6] | ***0.048*** | 8.7 [7.2-10.4] | 8.4 [6.9-9.9] | 9.6 [8.1-11.8] | ***<0.001*** |
| MV E/e` septal | 10.0 [8.0-13.1] | 10.1 [8.1-12.9] | 9.6 [7.8-13.5] | 0.818 | 10.3 [7.8-13.5] | 9.6 [7.5-12.8] | 13 [10.3-19.1] | ***<0.001*** |
| MV E/e` lateral | 7.7 [5.8-10.3] | 7.7 [5.7-9.9] | 8.1 [6.3-10.6] | 0.758 | 7.7 [5.6-11] | 7.1 [5.3-9.4] | 10.8 [7.6-15.2] | ***<0.001*** |
| MV E/A | 1.1 [0.9-1.3] | 1.1 [0.8-1.3] | 1.1 [0.8-1.4] | 0.317 | 1.1 [0.8-1.3] | 1.1 [0.8-1.3] | 1.2 [0.8-1.5] | 0.126 |

CAD- coronary artery disease, CV- chamber view, LV- left ventricular, LVEDD- left ventricular enddiastolic diameter, MI- myocardial infarction, MV- mitral valve, PAD- peripheral artery disease, RAVI- right atrial volume indexed to body surface area, RV- right ventricular, RVEDD- right ventricular enddiastolic diameter

**Supplemental Table 2: Regressions Analysis of Derivation Cohort**

| **Parameter** | **Complete Cohort**  (n=235) | | **Complete Cohort**  (n=235) | | **EAHsy-AF-Score** | | | |
| --- | --- | --- | --- | --- | --- | --- | --- | --- |
|  | **Univariate regression analysis** | | **Multivariate regression analysis** | |  |  |  |  |
|  | HR (95%CI) | p-value | HR (95%CI) | p-value | ***Parameter*** | ***ß-coefficient*** | ***OR (95% CI)*** | ***Resulting points*** |
| Age [years] | 1.06 (1.03-1.09) | ***<0.001*** | 5.02 (0,74-50.9) | 0.084 | Age  >75 yrs |  | 1.98 (1.04-3.77) | **1** |
| Hypertension | 3.57 (1.71-7.52) | ***<0.001*** | 0.684 (0.031-974.3) | 0.957 | Hypertension |  | 3.56 (1.69-7.52) | **1** |
| septal PATDI [ms] | 1.41 (1.22-1.64) | ***<0.001*** | 1.41 (1.21-1.64) | ***<0.001*** | sPA-TDI  >121 ms | 0.375 | 14.5 (5.63-37.24) | **4** |
| LAVI/a` | 1.83 (1.46-2.29) | ***0.001*** | 1.81 (1.43-2.31) | ***0.007*** | LAVI/a`  > 3.3 | 0.159 | 7.14 (3.38-15.11) | **2** |
| CAD | 2.22 (1.11-4.44) | ***0.024*** | 0.18 (0.01-2.68) | 0.212 |  |  |  |  |
| RAVI | 1.03 (1.0-1.05) | 0.051 |  |  |  |  |  |  |
| lateral PA-TDI [ms] | 1.17 (1.12-1.23) | ***<0.001*** |  |  |  |  |  |  |

**After testing for multicollinearities parameters for multivariable regression analysis were considered without lateral PA-TDI.**

AF- atrial fibrillation, CAD- coronary artery disease, CI- confidence interval, OR- odds ratio, LAVI- left atrial volume indexed to body surface area, septal PA-TDI- septal total atrial conduction time interval, RAVI- right atrial volume indexed to body surface area

**Supplemental Table 3: Predictors of AF in multivariable regressions analysis in the VALIDATION COHORT**

| **Parameter** | **Complete Cohort**  (n=290) | | **Complete Cohort**  (n=290) | |
| --- | --- | --- | --- | --- |
|  | **Univariate regression analysis** | | **Multivariate regression analysis** | |
|  | HR (95%CI) | p-value | HR (95%CI) | p-value |
| Age [years] | 1.078(1.052-1.105) | ***<0.001*** | 1.063 (0.964-1.171) | 0.221 |
| Height [cm] | 0.965 (0.94-0.991) | ***0.009*** | 0.929 (0.813-1.061) | 0.276 |
| Hypertension | 3.458 (1.813-6.596) | ***<0.001*** | 0.684 (0.001-374.127) | 0.906 |
| CHADS_2_ | 1.928 (1.44-2.582) | ***<0.001*** |  |  |
| CHADS_2_VA_2_Sc | 1.593 (1.324-1.916) | ***<0.001*** | 0.079 (0.001-7.326) | 0.272 |
| LAVI | 1.069 (1.043-1.097) | ***<0.001*** | 0.965 (0.818-1.139) | 0.677 |
| RAVI | 1.25 (1.121-1.394) | ***<0.001*** | 1.510 (0.59-3.866) | 0.391 |
| PA-TDI septal | 1.217 (1.15-1.288) | ***<0.001*** | 1.109 (0.99-1.232) | ***0.043*** |
| PA-TDI lateral | 1.045 (1.031-1.059) | ***<0.001*** | 0.904 (0.913-1.005) | 0.061 |
| LAVI/a` | 1.357 (1.195-1.54) | ***<0.001*** | 1.911 (1.203-3.036) | ***0.006*** |
| MV E/e`septal | 1.141 (1.078-1.208) | ***<0.001*** | 0.935 (0.483-1.81) | 0.842 |
| MV E/e`lateral | 1.122 (1.056-1.192) | ***<0.001*** | 0.832 (0.663-1.044) | 0.113 |

AF- atrial fibrillation, CI- confidence interval, HR- hazard ratio, LAVI- left atrial volume indexed to body surface area, MV- mitral valve, NT-pro-BNP- N-terminal pro-brain natriuretic peptide, PA-TDI- total atrial conduction time interval, RAVI- right atrial volume indexed to body surface area

**Supplemental Table 4: Individual thresholds of Scores predicting AF in the derivation and the validation Cohort**

|  | **Derivation Cohort**  **(n=235)** | | | | **Validation Cohort**  **(n=290)** | | | |
| --- | --- | --- | --- | --- | --- | --- | --- | --- |
| ***Score*** | *Cut-off* | *Sensitivity* | *Specifity* | *AUC* | *Cut-off* | *Sensitivity* | *Specifity* | *AUC* |
| ATLAS | 5.5 | 70.00 | 41.3 | 0.5848 | 7.5 | 65.08 | 55.36 | 0.603 |
| ARIC | 15.5 | 77.78 | 53.66 | 0.6198 | 16.5 | 68.25 | 59.82 | 0.557 |
| CHARGE AF | 5.1 | 68.57 | 53.38 | 0.6422 | 5.65 | 68.89 | 51.54 | 0.614 |
| CHADS_2_ | 2 | 37.9 | 77.2 | 0.6271 | 2 | 37.88 | 77.23 | 0.585 |
| EAHsy-AF | 4 | 98.5 | 93.3 | 0.9867 | 4 | 97.88 | 99.3 | 0.973 |

AUC- Area under the curve

**Supplemental Table 5: Mc Nemar test of predictional accuracy in the VALIDATION COHORT**

| **Patients with diagnosed AF** | | | | | | | | | | | | |
| --- | --- | --- | --- | --- | --- | --- | --- | --- | --- | --- | --- | --- |
|  | **CHADS_2_** | | | **CHARGE AF** | | | **ARIC** | | | **ATLAS** | | |
| **EAHsy-AF** | predicted no AF | predicted AF | p | predicted no AF | predicted AF | p | predicted no AF | predicted AF | p | predicted no AF | predicted AF | p |
| predicted no AF | 0 | 1 | ***<0.001*** | 0 | 1 | ***0.001*** | 0 | 1 | ***<0.001*** | 0 | 1 | ***<0.001*** |
| predicted AF | 41 | 24 |  | 14 | 30 |  | 18 | 44 |  | 17 | 45 |  |
| **Patients without diagnosed AF** | | | | | | | | | | | | |
| predicted no AF | 169 | 44 | ***<0.001*** | 64 | 57 | ***<0.001*** | 129 | 84 | ***<0.001*** | 126 | 87 | ***<0.001*** |
| predicted AF | 4 | 7 |  | 3 | 6 |  | 5 | 6 |  | 3 | 8 |  |

AF- Atrial fibrillation,
